# Use of spineless cactus associated with legume hay in the feedlot-finishing of lambs in semi-arid regions

**DOI:** 10.1371/journal.pone.0261554

**Published:** 2021-12-17

**Authors:** Maria Gabriela da Trindade Silva, Marcone Geraldo Costa, Mariana Campelo Medeiros, Gelson dos Santos Difante, Paulo Sérgio de Azevedo, Antonio Leandro Chaves Gurgel, João Virgínio Emerenciano Neto, Emmanuel Liévio de Lima Veras, Luís Carlos Vinhas Ítavo

**Affiliations:** 1 Academic Unit Specializing in Agricultural Science, Federal University of Rio Grande do Norte, Macaíba, Rio Grande do Norte, Brazil; 2 Faculty of Veterinary Medicine and Animal Science, Federal University of Mato Grosso do Sul, Campo Grande, Mato Grosso do Sul, Brazil; 3 Agricultural Science Campus, Federal University of Paraíba, Areia, Paraíba, Brazil; Tokat Gaziosmanpasa Universitesi, TURKEY

## Abstract

The objective of this study were to examine the effects of diets containing spineless cactus associated with hays of different legume species [gliricidia (*Gliricidia sepium*), “catingueira” (*Caesalpinia bracteosa*), “sabiá” (*Mimosa caesalpiniifolia Benth*) and “catanduva” (*Piptadenia moniliformis Benth*)] on the intake, digestibility, performance and carcass traits of lambs. Twenty-four ½ Santa Inês × ½ Soinga lambs with an average body weight of 21.4 ± 2.53 kg were distributed into four treatments in a completely randomized design. Treatments consisted of diets formulated with the association of spineless cactus (*Opuntia ficus-indica* Mill) and hay of four legume species, namely, “catanduva”, “sabiá” “catingueira” and gliricidia. The lowest intakes (P<0.05) of dry matter (DM), organic matter (OM), neutral detergent fiber (NDF) and total carbohydrates occurred in the animals that received the diet containing catingueira hay. However, there were no diet effects (P>0.05) on the intakes of crude protein (CP), ether extract and non-fibrous carbohydrates. The diets also did not change (P>0.05) the weight gain (0.197 kg/day) or final weight (33.18 kg) of the lambs or the apparent digestibility coefficients of DM, OM, CP and NDF. Consequently, the parameters of live weight at slaughter (34.10 kg), hot carcass weight (14.81 kg), cold carcass weight (14.66 kg), hot carcass yield (45.60%) and cold carcass yield (45.07%) exhibited the no response to treatment. In addition to these variables, the diets also did not influence subcutaneous fat thickness (2.54 mm), *longissimus* muscle area (13.34 cm^2^), morphometric measurements of the carcass, or the proportions of muscle and fat. Therefore, legume hays associated with spineless cactus can be used to feed ½ Santa Inês × ½ Soinga lambs in the feedlot, as this strategy provides heavy animals at the time of slaughter and carcasses with desirable degrees of muscularity and adiposity.

## Introduction

Semi-arid regions cover approximately 15% of the Earth’s surface and are home to around 14% of the global population [1]. Agricultural is the main economic activity held in these regions [2]. However, the irregular distribution of rainfall through time and space (Huang *et al*. 2016) is an obstacle to animal production. In this scenario, the use of biological elements (plant and animal) compatible with and adapted to the semi-arid environment is an alternative to produce food for the vulnerable populations inhabiting these areas [3, 4].

Sheep native to northeast Brazil, such as the Santa Inês and Soinga breeds, have advantages over larger ruminants, as they are adapted to adverse climatic conditions [5]. In the finishing of these animals, farmers have adopted feedlotting as a way to reduce slaughter age and obtain carcasses with greater uniformity [6]. Nonetheless, the feed cost is one of the main limitations of this practice [7, 8]. Therefore, lower-cost alternative feedstuffs with properties of resistance to the drought periods characteristic of the semi-arid region must be sought.

Spineless cactus (*Opuntia fícus indica* Mill) is widely used as animal feed in semi-arid regions [9, 10] due to its adaptability to these environments. This cactus can be supplied to animals fresh [4] or in the form of silage [10], constituting an important source of energy, water [4, 11] and non-fibrous carbohydrates, which include galactose, arabinose, xylose, fructose and, mainly, pectin [12]. However, spineless cactus has low crude protein and neutral detergent fiber contents [4], which can be corrected with the use of available legumes adapted to semi-arid regions.

When used in the form of hay, legumes can be a strategic reserve of fiber and protein for prolonged periods of drought [13]. Some of the main legumes used in the Brazilian semi-arid region are gliricidia (*Gliricidia sepium*), “catingueira” (*Caesalpinia bracteosa*), “sabiá” (*Mimosa caesalpiniifolia Benth*) and “catanduva” (*Piptadenia moniliformis Benth*), due to important characteristics for animal production such as perenniality, high crude protein content (>16%) and average ruminal dry matter degradability of 50–60% [14, 15].

Studies that investigate the association of spineless cactus with hays of legumes adapted to semi-arid conditions in the diet of sheep are scarce. Thus, further research on this subject is warranted, given the nutritional potential of these feedstuffs. In the present study, we raised the hypothesis that diets consisting of spineless cactus as an energy source plus legume hays (gliricidia, catingueira, sabiá and catanduva) as protein and fiber sources, would support a high level of production for lambs ½ Santa Inês × ½ Soinga, as a result, heavy and uniform carcasses.

This study were thus undertaken to examine the effects of diets containing spineless cactus associated with different legume hays (gliricidia, catingueira, sabiá and catanduva) on intake, digestibility, performance and carcass traits of ½ Santa Inês × ½ Soinga lambs.

## Materials and methods

All procedures conducted in this experiment complied with the guidelines established in Normative Resolution no. 25, of September 29, 2015, of the National Council for the Control of Animal Experimentation. The experiment was conducted in accordance with the norms set forth by the Ethics Committee on Animals Use—CEUA/UFRN (approval no. 055/2015).

### Experiment site

The experiment was conducted at the Academic Unit Specialized in Agricultural Sciences at the Agricultural School of Jundiaí, Federal University of Rio Grande do Norte, located in Macaíba—RN, Brazil (5°53 ’35.12" S and 35°21’ 47.03" W, 60 m above sea level). The experimental period was 60 days. The climate of the region is characterized as a dry sub-humid type, with water surplus occurring from May to August [16]. Average annual precipitation is 1048 mm and the potential cumulative average annual evapotranspiration is 1472 mm, according to the National Institute of Meteorology [17].

### Experimental design and animals

Experimental treatments were assigned as a completely randomized design. Treatments consisted of diets formulated with the association of spineless cactus (*Opuntia ficus-indica* Mill) and hay of four legume species (Table 1), namely, “catanduva” (*Piptadenia moniliformis*), “sabiá” (*Mimosa caesalpinifolia*), “catingueira” (*Caesalpina bracteosa*) and gliricidia (*Gliricidia sepium*). To make the legume hays, the manual harvesting of tender branches (≤8 mm thick) with leaves was performed. the material was then chopped to produce an average particle size of two cm, spread on plastic canvas in the sun and turned over every 30 min. After two days, the hay was bagged and stored in a moisture-free environment.

**Table 1:** Percentage and chemical compositions of the experimental diets (g/kg), on a dry-matter basis.

Ingredient (%)	Diet (hay)			
	Catanduva	Sabiá	Catingueira	Gliricidia
Spineless cactus	43.0	43.0	43.0	47.6
Catanduva hay	33.0	-	-	-
Sabiá hay	-	32.7	-	-
Catingueira hay	-	-	31.2	-
Gliricidia hay	-	-	-	33.0
Soybean meal	24.0	24.3	24.8	19.4
---------------------------------Chemical composition (g/kg DM) ---------------------------------				
Dry matter^1^	560.3	561.9	554.6	522.9
Ash	74.0	80.0	79.8	87.7
Crude protein	170.8	172.3	166.9	169.8
Ether extract	11.9	11.9	12.3	13.5
NDF	433.2	434.1	396.1	470.0
ADF	234.4	276.8	276.4	227.3
Lignin	132.6	132.6	122.3	87.9
NFC	310.0	310.4	334.8	336.7
TC	743.3	744.5	731.0	729.0
TDN	559.3	561.7	572.1	598.0

^1^g/kg as fed; DM = dry matter; NDF = neutral detergent fiber; ADF = acid detergent fiber; NFC = non-fibrous carbohydrates; TC = total carbohydrates; TDN = total digestible nutrients.

Twenty-four male lambs ½ Santa Inês × ½ Soinga (six animals per treatment) were used, with average initial live weight of 21.4 ± 2.53 kg, average age of 4.0 ± 1.0 months and from simple births. All animals received the same nutritional and health management in the phases prior to the finishing period. Before the beginning of the experiment, the animals were identified with numbered tags, administered an anthelmintic (7.5 mg/kg orally of levamisole hydrochloride at 5%), weighed and penned in individual stalls (1.0 ×1.5 m), which were equipped with a drinker and a feeder. The animals were allowed an eight-day period of adaptation to the facilities and management.

### Diets and dietary management

The diets were isonitrogenous and balanced to meet the nutritional requirements for a weight gain of 150 g/day (Table 1), according to NRC recommendations [18]. Due to the differences in CP content between the hays, soybean meal was used to adjust the level of this nutrient. With the purpose of reducing the selection of the ingredients of the diets by the animals, the bulky and concentrated foods were individually weighed and homogenized in plastic bags prior to the provision of the experimental diets. Therefore, feed was supplied as a complete mixture twice daily, 50% in the morning and 50% in the afternoon. Intake was monitored daily to keep orts around 10% of the total supplied and allow voluntary intake.

### Intake and digestibility

Dry matter intake (DMI) was calculated daily as the difference between the total feed supplied and orts. After 50 days of the experiment, a digestibility trial was conducted where the samples of the feed supplied, orts and feces were collected for three consecutive days and frozen for further analysis. To estimate fecal output, indigestible acid detergent fiber (iADF) was used as an internal marker [19]. The animal used to determine the iADF was a male a fistulated adult, male, castrated Morada Nova sheep, which received the same experimental diet for five days. After the period of acclimation to the diet, the diet ingredients, orts and feces were incubated *in situ* for 264 h [20]. The residue obtained after the incubation period was considered iADF. Fecal output was estimated by the following formula:

$$Fecal\;output\;(gDM/day)=\frac{Marker\;intake\;(g/day)}{Marker\;concentration\;in\;feces\;(g/gDM)}$$

Food samples, leftovers and feces were thawed and homogenized before pre-drying. An sub-sample was then removed and pre-dried in a forced-air oven at 55°C for approximately 72 h. Subsequently, were ground using 1-mm sieves for subsequent chemical analysis following the methodology of AOAC [21], to determine the dry matter (DM, method 2001.12), mineral matter (MM, method 942.05), crude protein (CP, method 984.13), lignin (method 973.18), ether extract (EE, method 920.39), neutral fiber (NDF, method 2002.04) and acid detergent fiber (ADF, method 973.18) contents. The organic matter (OM) content was calculated by the following equation: OM (g/kg) = 1000 –MM (g/kg).

Non-fibrous carbohydrates (NFC) were calculated by the following equation [22]: NFC (%) = 100 - (NDF + CP + EE + MM). The digestibility coefficients (DC) of DM and nutrients from the diets were calculated as the difference between the amounts of each nutrient ingested and excreted in the feces, using the following formula: DC (%) = [nutrient intake (g)—nutrient in feces (g)/nutrient intake (g)] × 100. Nutrient intake was determined as the total amount of a given nutrient supplied minus the amount of said nutrient in orts.

### Animal performance

To monitor weight gain, the animals were weighed every seven days, after a 12-h fast of solid feed, from the start of the experiment until the day of slaughter. Average daily weight gain (ADG) was calculated as the difference in weight between the first and last weeks divided by the number of days in the experimental period. Using the weight gain and DMI data, feed conversion [FC = (DMI × 60)/total weight gain] and feed efficiency [FE (%) = (total weight gain/(DMI × 60)) × 100] were calculated.

### Slaughter and on-carcass assessment

After the 60 experimental days, the lambs were deprived of solids and liquids for 12 h prior to slaughter. Immediately before slaughter, were weighed to determine the slaughter weight (SW).

At the time of slaughter, the animals were stunned in the atlanto-occipital region, which was followed by exsanguination by carotid and jugular section. After skinning and evisceration, the gastrointestinal tract (GIT) was weighed full and empty to determine the empty body weight (EBW). Then, the head (section at the atlanto-occipital joint) and extremities (section at the metacarpal and metatarsal joints) were removed. The carcasses were weighed to calculate the hot carcass weight (HCW) and the hot carcass yield [HCY (%) = HCW/SW × 100]. Next, they were transferred to a cold room at 5°C, where they were kept for 24 h hung by the tendons on appropriate hooks at a distance of 17 cm between the tarsometatarsal joints.

After this period, the following morphometric measurements were taken: internal carcass length, chest depth, heart girth, rump circumference, leg length, thigh circumference, chest width and rump width [23]. After the carcasses were chilled, the kidneys, perirenal fat and tail were removed to determine the cold carcass weight (CCW). Subsequently, chilling loss [CL (%) = HCW—CCW/HCW × 100] and cold carcass yield [CCY (%) = HCW—CCW/SW × 100] were calculated.

In the left half carcasses, a cross-section was made between the 12th and 13th ribs to measure *longissimus* muscle area on the *Longissimus dorsi* muscle. The muscle’s outline was traced on a transparent plastic sheet and two straight lines were drawn on the image, one measuring the maximum distance from the muscle in the mid-lateral direction (measurement A) and the other perpendicular to the previous one (measurement B). *Longissimus* muscle area was then calculated by the formula: REA = [(A/2 × B/2) × π]. Also on the *Longissimus dorsi* muscle, subcutaneous fat thickness (SFT) was measured between the last thoracic and first lumbar vertebrae using a digital caliper.

Subsequently, the left half carcasses were weighed and subdivided into five anatomical regions, which were weighed individually [23]. Cuts were as follows: shoulder, neck, loin, leg and rib. The legs were dissected to separate the muscles, bones and subcutaneous, pelvic and intermuscular fats [24] to determine the tissue composition of the leg. After the separation of tissues, the leg muscularity index [25] was calculated using the weight of the five muscles surrounding the femur, i.e. *Biceps femoris*, *Semimembranosus*, *Semitendinosus*, *Adductor femoris* and *Quadriceps femoris*.

### Statistical analysis

Data were subjected to analysis of variance and, when necessary, means were compared by Tukey’s test at 5% probability, using the PROC GLM command of the SAS statistical package (SAS University Edition, Sas Institute Inc. Cary, CA, USA). The following statistical model was applied: Yij = μ + ti + eij, where Yij = observed value of treatment i in replicate j; μ = overall mean; ti = treatment effect (i = catanduva hay, sabiá hay, catingueira hay, or gliricidia hay); and eij = random error associated with each observation.

## Results

### Nutrient intake and animal performance

There were differences between the treatment groups (P<0.05) for the intakes of DM (kg/day and %LW), OM, NDF and TC. Highest DM intakes were observed in the lambs that received the diet containing catanduva hay, the lowest in those fed catingueira hay, and intermediate for in the animals that consumed sabiá and gliricidia hays (Table 2). However, there were no diet effects (P>0.05) on the intakes of CP, EE or NFC. The diets also did not change (P>0.05) the weight gain (daily and total), final weight, FC or FE of the lambs (Table 2).

**Table 2:** Nutrient intake and performance of ½ Santa Inês × ½ Soinga lambs fed spineless cactus associated with legume hays.

Variable	Diet (hay)				CV (%)	P-value
	Catanduva	Sabiá	Catingueira	Gliricidia		
DM intake (kg/day)	1.34^a^	1.24^a^^b^	1.08^b^	1.18^a^^b^	10.24	<0.0001
DM intake (% LW)	4.02^a^	3.69^a^^b^	3.29^b^	3.58^a^^b^	8.74	<0.0001
OM intake (kg/day)	1.22^a^	1.12^a^^b^	0.98^b^	1.06^a^^b^	10.33	<0.0001
CP intake (kg/day)	0.228	0.218	0.192	0.227	13.63	0.0891
EE intake (kg/day)	0.015	0.015	0.012	0.018	30.77	0.6431
NDF intake (kg/day)	0.528^a^	0.497^a^^b^	0.397^b^	0.432^a^^b^	12.23	<0.0001
TC (kg/day)	0.972^a^	0.888^a^^b^	0.783^b^	0.825^a^^b^	10.15	<0.0001
NFC (kg/day)	0.443	0.393	0.397	0.425	9.27	0.8370
TWG (kg)	12.9	11.82	11.42	11.05	22.23	0.6482
ADG (kg/day)	0.215	0.197	0.190	0.184	22.21	0.6493
Final weight (kg)	33.60	33.53	32.65	32.95	8.20	0.8235
FC	6.54	6.67	6.17	6.54	25.45	0.9361
FE (%)	15.90	15.69	17.20	15.36	21.40	0.7429

DM: dry matter; OM: organic matter; CP: crude protein; EE: ether extract; NDF: neutral detergent fiber; TC: total carbohydrates; NFC: non-fibrous carbohydrates; TWG: total weight gain; ADG: average daily gain; FC: feed conversion; FE: feed efficiency; CV: coefficient of variation.

^a,b^Means followed by different letters in the rows differ statistically by Tukey’s test at 5% probability.

### Digestibility

The roughage source did not influence (P>0.05) the apparent digestibility coefficients of DM, OM, CP or NDF, which averaged 66.97%, 70.92%, 74.58% and 52.60%, respectively (Table 3).

**Table 3:** Apparent digestibility coefficients of dry matter (DM), organic matter (OM), crude protein (CP) and neutral detergent fiber (NDF) in ½ Santa Inês × ½ Soinga lambs fed spineless cactus associated with legume hays.

Variable	Diet (hay)				CV (%)	P-value
	Catanduva	Sabiá	Catingueira	Gliricidia		
DM (%)	70.30	69.38	64.69	63.50	6.94	0.3271
OM (%)	72.95	72.68	69.51	68.54	5.86	0.2547
CP (%)	70.80	76.60	74.87	76.05	4.03	0.5423
NDF (%)	50.17	54.88	48.85	56.49	9.07	0.0631

CV: coefficient of variation. ^a,b^Means followed by different letters in the rows differ statistically by Tukey’s test at 5% probability.

### Carcass traits

Roughage source did not affect carcass variables measured (Table 4). However, there were differences (P<0.05) between the treatment groups for EBW, with highest values observed in animals that received diets containing sabiá and catingueira hays and the lowest in those fed gliricidia hay. Intermediate EBW values were found in the lambs that consumed catanduva hay (Table 4). The group fed sabiá hay exhibited the highest GIT content, whereas catingueira hay provided the lowest value for this variable and no difference were detected (P>0.05) between the latter group and the animals that received the other hays (Table 4).

**Table 4:** Carcass traits of ½ Santa Inês × ½ Soinga lambs fed spineless cactus associated with legume hays.

Variable	Diet (hay)				CV (%)	P-value
	Catanduva	Sabiá	Catingueira	Gliricidia		
SW (kg)	31.95	33.96	30.91	33.15	6.51	0.2538
ECW (kg)	23.78^a^^b^	24.45^a^	24.35^a^	21.90^b^	5.91	<0.0001
GITC (kg)	5.67^a^^b^	6.65^a^	4.77^b^	5.53^a^^b^	11.37	0.0032
HCW (kg)	14.28	14.93	14.51	15.53	8.48	0.5361
CCW (kg)	14.13	14.65	14.35	15.30	8.53	0.6321
HCY (%)	44.68	43.97	46.93	46.84	4.81	0.5147
CCY (%)	44.26	43.48	46.40	46.14	5.24	0.3124
LMA (cm^2^)	11.28	8.84	10.44	10.80	28.37	0.2314
SFT (mm)	2.21	2.71	2.36	2.86	48.41	0.1370
CL (%)	1.08	1.83	1.16	1.50	90.21	0.7489

SW: slaughter weight; EBW: empty body weight; GITC: gastrointestinal tract content; HCW: hot carcass weight; CCW: cold carcass weight; HCY: hot carcass yield; CCY: cold carcass yield; CL: chilling loss; LMA: *Longissimus* muscle area; SFT: subcutaneous fat thickness; CV: coefficient of variation.

^a,b^Means followed by different letters in the rows differ statistically by Tukey’s test at 5% probability.

There were no roughage source effects (P>0.05) for any carcass length measurements except heart girth. Heart girth were highest in the lambs that consumed sabiá hay (Table 5).

**Table 5:** Morphometric measurements of the carcass of ½ Santa Inês × ½ Soinga lambs fed spineless cactus associated with legume hay.

Variable	Diet (hay)				CV (%)	P-value
	Catanduva	Sabiá	Catingueira	Gliricidia		
ICL (cm)	53.00	55.66	54.83	53.33	5.52	0.5342
Chest depth (cm)	21.50	22.83	22.50	21.83	7.98	0.4517
Heart girth (cm)	63.66^b^	68.50^a^	65.83^ab^	66.50^ab^	4.23	0.0021
Rump circumference (cm)	56.83	56.83	57.50	56.66	6.18	0.2915
Thigh circumference (cm)	41.50	41.50	42.00	43.50	6.51	0.1823
Chest width (cm)	22.50	24.66	22.33	22.83	12.88	0.1643
Rump width (cm)	20.66	21.50	22.33	22.83	12.80	0.3322
Leg length (cm)	38.50	38.50	37.83	37.33	3.17	0.2601

ICL: internal carcass length; CV: coefficient of variation.

^a,b^Means followed by different letters in the rows differ statistically by Tukey’s test at 5% probability.

Neck, leg and rib weights did not differ (P>0.05) between the treatment groups (Table 6). However, the lambs that consumed catanduva hay had the heaviest shoulder and the group fed gliricidia hay the lightest, whereas the other hays provided intermediate shoulder weights. Loin weight differed between the groups fed gliricidia and catingueira hays, which exhibited the highest and lowest values, respectively, while intermediate loin weights were found in the animals fed the other two diets.

**Table 6:** Weight of primal cuts of ½ Santa Inês × ½ Soinga lambs fed spineless cactus associated with legume hays.

Weight (kg)	Diet (hay)				CV (%)	P-value
	Catanduva	Sabiá	Catingueira	Gliricidia		
Shoulder	1.23^a^	1.17^a^^b^	1.22^a^^b^	1.10^b^	7.54	0.0002
Neck	0.71	0.71	0.68	0.82	19.19	0.1532
Loin	1.19^b^	1.22^a^^b^	1.17^b^	1.39^a^	9.31	<0.0001
Leg	2.23	2.19	2.25	2.16	8.33	0.6732
Rib	1.79	1.91	1.95	1.96	14.87	0.1670

CV: coefficient of variation.

^a,b^Means followed by different letters in the rows differ statistically by Tukey’s test at 5% probability.

Source of roughage did not impact reconstituted leg tissue proportions with the exception of bone percentage (Table 7). Thus, the legs of lambs fed catanduva and sabiá hays had a higher bone percentage (P<0.05) than those of lambs fed gliricidia hay. The amount of bone in the leg of lambs that received catingueira hay were similar to the others. Muscle:bone ratio in the leg differed between the groups fed gliricidia and sabiá hays, which showed the highest and lowest values, respectively, whereas the other hays provided intermediate values.

**Table 7:** Average tissue composition of the leg of ½ Santa Inês × ½ Soinga lambs fed spineless cactus associated with legume hays.

Variable	Diet (hay)				CV (%)	P-value
	Catanduva	Sabiá	Catingueira	Gliricidia		
Reconstituted leg (kg)	2.16	2.05	2.09	2.11	9.86	0.2134
Bone (%)	18.98^a^	18.78^a^	17.52^ab^	14.54^a^	14.29	0.0341
Muscle (%)	66.70	61.06	62.61	59.23	10.88	0.4012
Fat (%)	10.09	12.71	11.56	17.07	39.08	0.3753
Muscle:bone	3.51^a^^b^	3.26^a^	3.63^a^^a^	4.17^a^	14.61	0.0210
Muscle:fat	6.68	6.26	5.95	3.97	38.40	0.0611
Bone:fat	1.93	1.94	1.68	1.00	43.56	0.5219
Leg muscularity index (g/cm)	0.11	0.10	0.11	0.10	12.18	0.0783

CV: coefficient of variation.

^a,b^Means followed by different letters in the rows differ statistically by Tukey’s test at 5% probability.

## Discussion

### Nutrient intake and animal performance

The lowest DMI, expressed both in kg/day and %LW, were observed in the animals fed the diet composed of the association of spineless cactus and catingueira hay. As a result, also had the lowest OM, NDF and TC intakes. Several factors can affect DM intake by herbivores, especially in ruminants. Baumont et al. [26] considers NDF as one of the main factors in controlling DM consumption by ruminants. However, in this work, the diet that received catingueira hay presented the lowest NDF content (396.1 g/kg DM) in (Table 1) and, even so, provided the lowest DM intake.

Two factors can explain this result. One of them would be the high concentration of condensed tannins present in catingueira leaves [27], since high tannin concentrations markedly reduced intake, changing the acceptability of a food [28]. Gonzaga Neto et al. [29] observed that the DMI of Morada Nova sheep linearly decreased due to the increased inclusion of catingueira hay in the animals’ diet. The authors attributed this response to the high tannin content (6.30%) of the hay, which exceeded the critical limit of 4.0% for sheep [30]. Another explanation would be the higher proportion of ADF in relation to NDF in the diet with catnigueira hay (70.67%) and, consequently, a lower proportion of hemicellulose, when compared to the other diets. The greater participation of the FDA in the NDF fraction may have been responsible for the trend (0.0631) of reduced NDF digestibility (Table 3).

The DM intake (in kg/day) observed in all treatment groups throughout the experimental period is in line with the 0.760 to 1.320 kg/day predicted by the NRC [18] for sheep in this category. It is also noteworthy that the intakes of CP and NFC remained constant among the animals that received the different diets (Table 2), due to the slight variation in concentration of these nutrients in the diets (Table 1). Therefore, it is possible that, regardless of the diet, the animals consumed nutrients in sufficient amounts to meet their requirements. For these reasons, weight gain (daily or total), final weight, FC and FE were not different.

In the present study, ADG were 0.197 kg/day, which exceeds the 0.150 kg/day initially estimated by the NRC [18]. The consumption and performance prediction equations developed by international committees are mostly obtained in temperate countries. Therefore, designed specifically for their own environmental characteristics, races and food compositions that are very contrasting with the reality found in tropical regions. As a result, there is an under or overestimation of the requirements of animals raised in tropical climate regions [31–33].

However, the results for weight gain observed allowed the animals to reach an average body weight of 33.18 kg at the end of the 60-day confinement period, a value above the 30 kg recommended by the sheep meat consumer market [34, 35]. This confirms the potential of using legume hays associated with spineless cacti for sheep in confinement in semiarid environments.

### Digestibility

The lack of a diet effects on the apparent digestibility coefficients of DM, OM, CP and NDF may be related to the similar intakes of NFC and CP between the treatment groups (Table 2), since a dietary imbalance, in terms of CP and NFC intake, can reduce the proliferation of ruminal microorganisms, thereby reducing digestibility [9]. Another factor that can reduce digestibility is the lipid content of the diet [36], but EE intake did not change (Table 2).

The average digestibility coefficients of DM, OM, CP and NDF found in this study are similar to those reported in experimental diets composed of maize, soybean meal and conventional roughages such as bermudagrass hay and maize silage [9, 36, 37]. What emerges is that the possible interference of tannins in the DM intake of the catingueira hay diet was not enough to reduce the digestibility of the diets. However, the high participation of the FDA in the NDF fraction may have been responsible for the tendency to reduce the fiber digestibility of the diet containing catinguera hay. This fact highlights the ability of neutral detergent fiber to promote greater ruminal filling and reduce the rate of forage disappearance in the rumen [38].

### Carcass traits

The results seen for HCW and CCW are possibly related to SW, which were similar between the animals. This similar SW were also reflected in the uniform HCY, CCY, REA, SFT (Table 4) and morphometric measurements of the carcass (Table 5). The intakes of CP and NFC were also not affected by the association of the spineless cactus with the legume hays, which also contributed to the similar carcass traits. The mean HCW and CCW of 14.81 and 14.66 kg, respectively, met the minimum values indicated by Silva Sobrinho [39] for the characterization of good-quality carcasses, i.e., HCW ≥ 14.40 kg and CCW ≥ 13.80 kg.

The observed differences for GIT content and EBW can be explained by the NDF intake of the lambs (Table 2), since higher consumption of this nutritional component is related to a longer residence time of the feed in the GIT [37]. Accordingly, the lambs fed the diet containing catingueira hay consumed a smaller amount of NDF and, consequently, had less GIT content and a heavier EBW. Given that fiber has a direct effect on volume on the capacity of rumen musculature [40].

The *longissimus* muscle area remained constant between the treatment groups and was consistent with the response shown by SW as well as with the HCW and CCW. The *Longissimus* muscle is directly related to the weight of the animal at the time of slaughter, and this muscle tends to be larger in bulkier carcasses [41, 42]. The average observed area of the *Longissimus* muscle of 13.34 cm^2^ exceeds the 11.60 cm^2^ reported by Silva et al. [37] and the 8.43 cm^2^ described by Urbano et al. [43] for the carcass of Santa Inês sheep finished in feedlot. It is important to emphasize that because the REA corresponds to the muscle of the carcass [3], the carcasses with higher REA are more valued.

Although SFT did not change in response to the diets, the values found were higher than those described in other studies with woolless sheep [44, 45]. These higher SFT may have been one of the reasons for the reduced chilling loss (1.14%), considering that a thicker layer of subcutaneous fat implies less chilling loss [46].

There was little variation in the weights of primal cuts. This finding is explained, in part, by SW and the amount of body fat, since carcasses with similar weights and fat contents have almost all body regions in similar proportions [3]. Based on the weights of primal cuts, we can infer that the carcasses provided satisfactory cut yields, considering that the most valuable cuts—leg, loin and shoulder [44]—account for about 60% of the total yield of cuts.

The average tissue composition of the leg revealed that the muscles had the highest contribution (66.70%), followed by bone (18.89%) and fat (10.09%). In addition, there was a variation between the diets only for the proportion of bone tissue. These tissue components have different orders of development, with the bone tissue growing earlier, followed by the muscle and, finally, the adipose tissue [45]. The lower proportion of bone tissue in animals that received Gliricidia hay may be associated with the lower numerical value observed for the proportion of muscle tissue. Because there is a high correlation between the amount of soft tissue and the proportion of bone in the carcass [47].

The association of spineless cactus with legume hays showed to be promising for use in the diet of feedlot sheep in semi-arid environments, as the animals’ weight gains were higher than the pre-established goals, which resulted in heavy animals at the time of slaughter and carcasses with desirable degrees of muscularity and adiposity.

Therefore, these results confirm the hypothesis that diets consisting of spineless cactus as an energy source plus hay from the legume species gliricidia, catingueira, sabiá and catanduva as a source of protein and fiber provide high productive performance in ½ Santa Inês × ½ Soinga lambs and, consequently, heavy and uniform carcasses. However, further research using these pulses associated with spineless cactus in different production systems will provide a better insight into the use of these diets in a larger number of animals, in different sexes and co-ages of animals. Understanding these relationships can contribute to the development of new nutritional strategies based on modifications in the concentrate: forage ratio in ruminant diets.

## Conclusions

Spineless cactus associated with hay of legume species such as catanduva (*Piptadenia moniliformis*), sabiá (*Mimosa caesalpinifolia*), catingueira (*Caesalpina bracteosa*) and gliricidia (*Gliricidia sepium*) can be used in the finishing of feedlot lambs in semi-arid regions, as they provide animals with desirable performance rates and carcass traits.
